# Implementation of a Shunt Insertion Protocol and Its Effect on Institutional Shunt Infection Rates

**DOI:** 10.7759/cureus.95531

**Published:** 2025-10-27

**Authors:** Diana Jovett Sanchez, Miriam Claire O Dy, Juan Silvestre G Pascual, Ronnie E Baticulon, Kathleen Joy O Khu

**Affiliations:** 1 Division of Neurosurgery, Department of Neurosciences, College of Medicine and Philippine General Hospital, University of the Philippines, Manila, PHL

**Keywords:** hydrocephalus, lmic, shunt infection, shunt protocol, ventriculoperitoneal shunt

## Abstract

Background

Hydrocephalus treatment often involves cerebrospinal fluid diversion via a ventriculoperitoneal shunt (VPS) or endoscopic third ventriculostomy. However, VPS is associated with higher postoperative complication rates, particularly infection. The Hydrocephalus Clinical Research Network (HCRN) has developed protocols to reduce shunt infection rates, demonstrating significant success. This study aimed to evaluate the effectiveness of a locally adapted shunt protocol in reducing shunt infection rates.

Methodology

An 11-step Philippine General Hospital (PGH) Shunt Protocol, modeled after the HCRN protocol, was implemented from January to June 2023. Data from 69 patients who underwent shunt procedures were analyzed. Patient characteristics, operative factors, protocol compliance, and postoperative outcomes were reviewed. The primary outcome measure was shunt infection. A simple logistic regression analysis was performed to determine the association between patient characteristics, surgical factors, and individual protocol steps with shunt infection.

Results

The overall shunt infection rate was 11.6% (8 out of 69 cases). None of the patient characteristics and operative variables were significantly associated with shunt infection risk. Full compliance with the protocol was observed in only 36 out of 69 (52%) cases, but protocol adherence did not significantly reduce infection risk. No individual step in the shunt protocol was identified to be significantly associated with decreasing shunt infection risk.

Conclusions

Given the suboptimal compliance of 52% (36 out of 69 cases), we were unable to draw any significant conclusions regarding the efficacy of the PGH Shunt Protocol. No factors were identified among patient characteristics and operative variables to be associated with shunt infection risk.

## Introduction

Hydrocephalus accounts for a significant portion of neurosurgical consults worldwide [1,2]. The etiology is varied, and it may be involved in multiple neurosurgical disease processes, the majority being congenital in nature [3]. The mainstay of treatment is cerebrospinal fluid (CSF) diversion, in the form of ventriculoperitoneal shunting (VPS) or endoscopic third ventriculostomy (ETV) [4]. Success rates are similar between ETV and VPS; however, postoperative complication rates are higher with VPS compared to ETV, at 27.1% and 4.6%, respectively [4].

Complications of VPS procedures include shunt infection and shunt malfunction [5]. Shunt infection rates vary across different institutions, countries, and patient populations. High-income countries (HICs) report an infection rate of 2-9% [6,7]; however, low- to middle-income countries (LMICs) have a higher reported rate of 8.6-50% [3]. Various techniques have been implemented to decrease shunt infection rates, such as improving surgical technique, using antibiotic-impregnated shunts, developing new antimicrobial materials, and optimizing perioperative care protocols [8].

The Hydrocephalus Clinical Research Network (HCRN) Protocol was developed in 2011 with the goal of reducing shunt infection rates. The protocol was initiated by the collaboration of pediatric neurosurgical centers in North America and was found to reduce shunt infection rates from 8.8% to 5.7% [8]. The 11-step HCRN protocol is as follows: (1) sign on operating room (OR) door restricting traffic; (2) position head away from the main OR door; (3) ask for antibiotics; (4) clip hair as needed; (5) dirt, debris, and adhesive material removed; (6) chlorhexidine gluconate and isopropyl alcohol (Chloraprep, Becton, Dickinson, and Company, USA) prep applied to the surgical field and not washed off; (7) hand scrub with betadine or chlorhexidine; (8) double glove (non-latex); (9) iodine impregnated incision drape (3M™ Ioban™, USA) application; (10) injection of vancomycin/gentamicin into shunt reservoir; and (11) one postoperative dose of the same antibiotic [8].

A second report by the HCRN decreased the steps from 11 to 5 and included antibiotic-impregnated catheters, resulting in similar infection rates [9]. The last and most recent iteration in 2022 included a simplified protocol with five core steps, emphasizing the importance of protocol compliance [10]. The five steps were the following: (1) intravenous antibiotic administration before skin incision; (2) use of chlorhexidine as final preparation; (3) hand scrub by all participants; (4) double gloves by all participants; and (5) application of Ioban (3M™ Ioban™, USA) to the surgical field [10]. This protocol further reduced the overall shunt infection rates from 5.7% to 5.1% [10].

Currently, there are no published standardized protocols in the Philippines to reduce shunt infection rates. In this study, we aimed to assess the effect of a shunt insertion protocol (Philippine General Hospital (PGH) Shunt Protocol) on our institution’s shunt infection rate, building on previous unpublished work. This study will add to the body of knowledge regarding the effectiveness of a shunt protocol in decreasing the rate of shunt infection.

## Materials and methods

This was a retrospective review of prospectively collected data. The study was approved by the University of the Philippines Manila Research Ethics Board (approval number: UPMREB 2024-0730-01) and the reporting followed the Strengthening the Reporting of Observational Studies in Epidemiology (STROBE) guidelines.

All pediatric and adult patients who underwent shunting operations from January 2023 to June 2023 were included. As adapted from the work of Kestle et al. [8], the shunting operations included ventriculoperitoneal, ventriculoatrial, ventriculopleural, or cystoperitoneal shunts. The procedures were further categorized into primary or first-time shunts, shunt revision (replacement of shunt parts), and secondary shunt insertion with or without infection (placement of a shunt after a temporary CSF diversion such as an external ventricular drain (EVD) or Ommaya reservoir). Patients who had an EVD or Ommaya reservoir insertion were not included.

**Figure 1:**
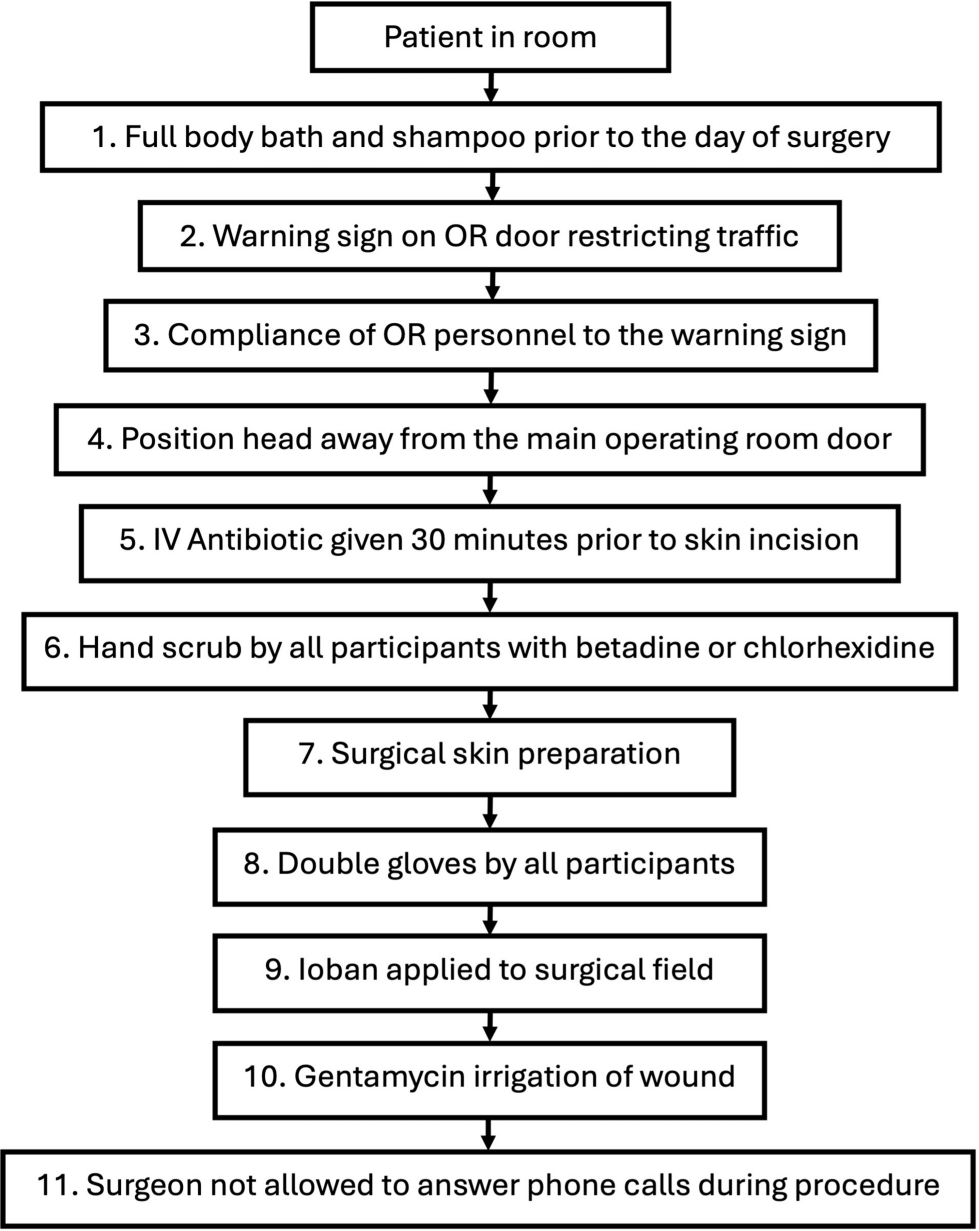
Philippine General Hospital (PGH) Shunt Protocol. The PGH Shunt Protocol was adapted from the 2011 Kestle et al. [8] Hydrocephalus Clinical Research Network Protocol. Permission has been acquired for modification and use of the protocol in correspondence with Dr. John Kestle. OR = operating room; IV = intravenous

The PGH Shunt Protocol was implemented for all shunting procedures during this time period. This was an 11-step protocol modeled after the HCRN Protocol [8], but some modifications were made to account for local institutional practices (Figure 1). In our protocol, gentamycin irrigation of the wound was added instead of vancomycin injection into the shunt reservoir because vancomycin is a regulated drug in our hospital and is not readily available for surgical use. Surgeons were also not allowed to answer calls while the surgery was ongoing to decrease the chances of contamination of the sterile field. The printed protocol was accomplished by the circulating nurse and counter checked by the surgeon at the end of every operation. The completed forms were collected, and the data were reviewed and tabulated.

Data on patient characteristics included age, sex, type of hydrocephalus, original pathology (congenital, infectious, vascular, trauma, tumor, others), a prior history of shunt infection or revision, and associated comorbid infection during the time of surgery. Intraoperative factors included the number of previous cranial surgeries, total number of previous surgeries, urgency of operation (emergency or elective), surgeon experience (junior or senior trainee), number of persons in the room, duration of surgery, type of surgery performed (primary, secondary, revision), use of endoscopy, and presence of EVD or Ommaya reservoir. Postoperative data included date of follow-up, time to shunt infection, presenting signs and symptoms of shunt infection, and CSF counts and cultures. The period of follow-up was one year postoperatively.

The outcome variable was shunt infection, defined by the HCRN as (1) identification of organisms on culture or Gram stain from CSF, wound swab, or pseudocyst fluid; (2) CSF pleocytosis obtained from a shunt tap associated with fever and/or shunt malfunction; (3) clinical signs of wound infection, including wound dehiscence, wound discharge, and shunt erosion; or (4) peritonitis in a patient with a ventriculoperitoneal or cystoperitoneal shunt and/or positive blood cultures in a child with a ventriculoatrial or ventriculopleural shunt [8,10].

To assess for association with shunt infection, a simple logistic regression analysis was performed for patient characteristics and intraoperative factors. Individual protocol factors were also analyzed via simple logistic regression to assess their association with shunt infection. The level of significance was set at 0.05. Data processing and analysis were performed using Stata 17 (StataCorp LLC, TX, USA).

## Results

A total of 69 patients were included in this study, eight of whom developed shunt infection, with an overall shunt infection rate of 11.6%. Patient characteristics and surgical factors are listed in Table 1. Some of the factors that were associated with greater odds of infection were age less than one year, patients with a previous history of shunt infection, emergency operations, secondary shunt surgery, surgery by junior trainees, and procedures performed with endoscopy, although the value did not reach statistical significance (Tables 2, 3).

**Table 1:** Patient demographics and clinical characteristics (n = 69). OR = operating room; IQR = interquartile range

Characteristics	Frequency (%)
Age	
<1 year	26 (38%)
1–18 years	20 (29%)
>18 years	23 (33%)
Sex	
Male	29 (42%)
Female	40 (58%)
Entry	
Emergency room	50 (72%)
Elective	19 (28%)
Shunt history	
History of shunt infection	2 (3%)
History of shunt revision	5 (7%)
Pathology	
Congenital	30 (43%)
Tumor	17 (25%)
Infectious	13 (19%)
Post-hemorrhagic	7 (10%)
Post-traumatic	2 (3%)
Type of hydrocephalus	
Non-communicating	50 (72%)
Communicating	19 (28%)
Comorbid infection	40 (58%)
Respiratory	23 (58%)
Central nervous system	18 (45%)
Bacteremia/Sepsis	17 (42%)
Genitourinary	6 (15%)
Tuberculosis	6 (15%)
Previous surgery, count, median, (IQR)	1 (0–2)
Previous cranial surgery, count, median (IQR)	0 (0–2)
Classification of operation	
Elective	21 (30%)
Emergency	48 (70%)
Type of surgery	
Primary	51 (74%)
Revision	5 (7%)
Secondary (no infection)	8 (12%)
Secondary (with infection)	5 (7%)
Surgeon experience	
Junior trainee	37 (54%)
Senior trainee	32 (46%)
Surgery with endoscopy use	5 (7%)
Surgery duration, minutes, median (IQR)	89 (65–122)
Persons inside the OR, count, media (IQR)	5 (5–6)

**Table 2:** Clinical factors associated with shunt infection (n = 69). *: OR cannot be determined due to zero cases among exposed (history of shunt revision). OR = odds ratio; CI = confidence interval

Clinical factors	Frequency (%)		Adjusted OR	95% CI	P-value
	With shunt infection	Without shunt infection			
	n = 8	n = 61			
Age					
<1 year	5 (19.23%)	21 (80.77%)	5.24	0.56, 48.65	0.145
1–18 years	2 (10.00%)	18 (90.00%)	2.44	0.20, 29.19	0.48
>18 years	1 (4.35%)	22 (95.65%)	Reference		
History of shunt infection					
With	1 (50.00%)	1 (50.00%)	8.57	0.48, 152.73	0.144
Without	7 (10.45%)	60 (89.55%)	Reference		
History of shunt revision					
With	0 (0.00%)	5 (100.00%)	*		
Without	8 (12.50%)	56 (87.50%)			
Pathology					
Congenital	5 (16.67%)	25 (83.33%)	2.4	0.53, 10.97	0.259
Other pathologies	3 (7.69%)	36 (92.31%)	1.69	0.36, 7.88	0.506
Type of hydrocephalus					
Communicating	3 (15.79%)	16 (84.21%)	Reference		
Non-communicating	5 (10.00%)	45 (90.0%)	0.59	0.13, 2.77	0.506
Comorbid infections					
With	5 (12.50%)	35 (87.50%)	1.24	0.27, 5.65	0.783
Without	3 (10.34%)	26 (89.66%)	Reference		
Number of previous cranial surgeries					
<2 surgeries	5 (9.80%)	46 (90.20%)	Reference		
≥2 surgeries	3 (16.67%)	15 (83.33%)	1.84	0.39, 8.63	0.439

**Table 3:** Surgical factors associated with shunt infection (n = 69). OR = odds ratio; CI = confidence interval

Operative factors	Frequency (%)		Adjusted OR	95% CI	P-value
	With shunt infection	Without shunt infection			
	n = 8	n = 61			
Operation done					
Elective	1 (4.76%)	20 (95.24%)	Reference		
Emergency	7 (14.58%)	41 (85.42%)	3.41	0.39, 29.68	0.266
Type of surgery					
Primary	4 (7.84%)	47 (92.16%)	Reference		
Revision	1 (20.00%)	4 (80.00%)	2.94	0.26, 32.95	0.382
Secondary (no infection)	2 (25.00%)	6 (75.00%)	3.92	0.59, 26.14	0.159
Secondary (with infection)	1 (20.00%)	4 (80.00%)	2.94	0.26, 32.95	0.382
Surgeon experience					
Junior	7 (18.92%)	30 (81.08%)	7.23	0.84, 62.38	0.072
Senior	1 (3.12%)	31 (96.88%)	Reference		
Duration of operation					
<90 minutes	5 (14.29%)	30 (85.71%)	Reference		
≥90 minutes	3 (8.82%)	31 (91.18%)	0.58	0.13, 2.65	0.482
Number of persons in the operating room					
≤4 persons	4 (26.67%)	11 (73.33%)	Reference		
>4 persons	4 (7.41%)	50 (92.59%)	0.22	0.05, 1.02	0.053
Endoscopy					
Done	2 (40.00%)	3 (60.00%)	6.44	0.89, 46.53	0.065
Not done	6 (9.38%)	58 (90.62%)	Reference		
Compliance with the shunt protocol					
Complied	3 (8.33%)	33 (91.67%)	0.51	0.11, 2.32	0.383
Deviated	5 (15.15%)	28 (84.85%)	Reference		

Demographics

A total of 69 procedures were performed on 69 different patients. Patients younger than one year comprised the largest age group (n = 26, 38%), followed by adults older than 18 years (n = 23, 33%), and children/adolescents aged 1-18 years (n = 20, 29%). Females accounted for 58% (n = 40), while males comprised 42% (n = 29). Our results showed that in terms of age, patients aged less than one year old had five times higher odds of having shunt infection compared to adults (p = 0.145; 95% confidence interval (CI) 0.56, 48.56).

Shunt history

Two patients had a previous shunt infection (3%), while five (7%) patients had a history of shunt revision. Our results showed that a history of shunt infection increased the odds of developing another shunt infection by 8.5 times compared to those who did not have any history of shunt infection (p = 0.144; 95% CI = 0.48, 152.73).

Primary pathology and comorbid infections

The most common pathology was congenital, comprising almost half of the patients (n = 30, 43%), followed by tumor-associated (n = 17, 25%), infectious (n = 13, 19%), post-hemorrhagic (n = 7, 10%), and post-traumatic (n = 2, 3%) hydrocephalus. A congenital pathology showed a 2.4 times higher odds of developing shunt infection compared with other pathologies (p = 0.259, 95% CI = 0.53, 10.97). The majority of hydrocephalus cases were non-communicating (n = 50, 72%). The most commonly seen comorbid infections were respiratory (n = 23, 58%), followed by central nervous system infections (n = 18, 45%) and sepsis (n = 17, 42%).

Surgical factors

Nearly three-quarters of the cases (n = 48, 70%) were performed in an emergency setting, while elective surgeries comprised 30% (n = 21). Emergency operations were associated with a 3.4-fold increased association with developing shunt infection compared to elective shunting procedures (p = 0.266, 95% CI = 0.39, 29.68). Among the 69 procedures performed, 74% were primary shunt insertions (n = 51), 12% were secondary insertions without infection (n = 8), 7% were revision surgeries (n = 5), and 7% were secondary insertions with infection (n = 5). Both revision and secondary shunt surgeries increased the risk of infection by two to three times compared with primary procedures. Endoscopic procedures, performed in 7% (n = 5) of cases, were associated with a 6.4-fold higher infection risk (p = 0.065; 95% CI = 0.89, 46.53).

More than half of the procedures (n = 37, 54%) were performed by junior residents (fourth year and below). Surgeries performed by junior residents had 7.2 times higher odds of infection compared with those by senior residents (fifth and sixth year) (p = 0.072, 95% CI = 0.84, 62.38). The median operation duration was 89 minutes, and the median number of persons inside the OR was 5.

Shunt protocol compliance

Full compliance with the shunt protocol was seen in 36 out of 69 cases (52% compliance rate). There was only partial compliance in the rest: 25 cases followed 10/11 steps, six cases followed 9/11 steps, and two cases followed 8/11 steps. All cases complied with three steps: hand scrubbing with betadine or chlorhexidine, surgical skin preparation, and gentamicin wound irrigation. The step with the lowest adherence rate was the application of Ioban (3M™, Ioban™, USA) to the surgical field, mainly due to surgeon preference. This was followed by preoperative full-body bathing and placement of a warning sign in the OR to restrict traffic, respectively. The low compliance with preoperative bathing was due to the emergency nature of the majority of shunting operations, with patients admitted directly from the emergency department, where bathing facilities were limited. The inability to place a warning sign on the OR door was due to staffing concerns; circulator nurses were frequently assigned to more than one OR per shift, making it difficult to consistently place the signage and enforce traffic restrictions. There was no individual step in the shunt protocol that was identified to be significantly associated with decreasing shunt infection risk, but the application of Ioban appeared to be protective (odds ratio = 0.24, p = 0.07, 95% CI = 0.05, 1.12) (Table 4).

**Table 4:** Association of complied steps of the shunt protocol with shunt infection (n = 69). *: OR cannot be determined due to zero cases among unexposed (non-compliant with the step). OR = odds ratio; CI = confidence interval; IV = intravenous

Clinical factors		Frequency (%)		OR	95% CI	P-value
	Compliance	With shunt infection (n = 8)	Without shunt infection (n = 61)			
Full body bath	59 (85.51%)	7 (11.86%)	52 (88.14%)	1.21	0.13, 11.06	0.865
Sign on the door restricting traffic	62 (89.86%)	8 (12.90%)	54 (87.10%)	*		
Compliance of personnel with the warning sign	67 (97.10%)	8 (11.94%)	59 (88.06%)	*		
Head away from the main door	68 (98.55%)	8 (11.76%)	60 (88.24%)	*		
IV antibiotics 30 minutes prior	66 (95.65%)	8 (12.12%)	58 (87.88%)	*		
Hand scrub by all participants	69 (100.00%)	8 (11.59%)	61 (88.41%)	*		
Surgical skin preparation	69 (100.00%)	8 (11.59%)	61 (88.41%)	*		
Double gloves	68 (98.55%)	8 (11.76%)	60 (88.24%)	*		
Ioban application	53 (76.81%)	4 (7.55%)	49 (92.45%)	0.24	0.05, 1.12	0.070
Gentamicin irrigation of the wound	69 (100.00%)	8 (11.59%)	61 (88.41%)	*		
No calls	66 (95.65%)	8 (12.12%)	58 (87.88%)	*		

Shunt infection: microorganisms and antibiotics

In total, eight patients developed shunt infection, and only five had growth on CSF cultures. The most common isolates were *Klebsiella pneumoniae* and *Staphylococcus aureus*. The antibiotic coverage was culture-guided, and the most common antibiotics used were vancomycin, meropenem, ceftazidime, oxacillin, and amikacin. All patients underwent secondary shunt insertion after completion of antibiotics.

## Discussion

The overall shunt infection rate after protocol institution was 11.6%, which was comparable to other LMICs that reported shunt infection rates of 8.6%-50% [11]. However, this figure compared negatively with previous local data. The PGH Shunt Protocol was first instituted in 2019, with a decrease in shunt infection rate from 11.9% to 7.7% over a nine-month period from July 2019 to March 2020 (personal communication). The protocol was suspended during the COVID-19 pandemic, when other protocols were instituted. It was only resumed in 2023. While there was no statistically significant factor associated with shunt infection in our data, certain patient characteristics and operative variables merit attention, as they have been reported as significant in previously published studies.

Patient-related factors

Data on a specific age cutoff for increased shunt infection risk vary among different studies. Age of less than six months to one year was found to be a significant factor in increasing the risk of infection [12]. Although it was not statistically significant in our results, patients younger than one year exhibited fivefold higher odds of developing shunt infection compared to those older than 18 years. The increased infection risk may be due to a number of contributing factors. First, the innate immunity of a child is immature [13]. Cells needed during the first line of defense to infection have inadequate chemotaxis, and inflammatory mediators are compromised during this stage of life; hence, the diminished febrile response in newborns as well as the different response to wound healing [13,14]. Further, the cellular and humoral immunity are underdeveloped in those aged less than one year. During this stage, children rely on existing IgG obtained from placental transfer and only develop immunity similar to adults by four to six years of age [14]. Hence, the ability to produce antibodies and repel infection is insufficient in younger patients.

A prior history of shunt infection and shunt revision was found to be associated with shunt infection risk, in line with previous literature [15,16]. We found a non-significant, eightfold increase in the odds of developing another shunt infection among patients with prior shunt infection. They appear to be associated with antibiotic resistance, biofilm formation, and an increased risk of shunt infection with each surgical intervention [17]. Similar mechanisms may cause the increased risk observed in patients with a prior history of cranial surgeries [18]. Comorbid infections such as urinary tract and respiratory infections, presence of a gastrostomy tube, and cardiac comorbidities were also identified to be significant risk factors for shunt infection [11].

The etiology and type of hydrocephalus also appeared to influence shunt infection risk, with congenital hydrocephalus and those associated with spinal dysraphism identified to be risk factors for shunt infection [12]. In our results, congenital pathology had a 2.4-fold higher association with shunt infection compared with other pathologies. This was postulated to be due to the younger patient age in this subset, suggesting that age itself may be the more critical determinant [19].

Surgical factors

Emergency procedures accounted for 70% of cases and showed more than threefold higher odds of infection compared to elective surgeries. Factors linked to increased risk include (1) site of the operation may not be a designated neurosurgical theater; hence, the bacterial load in the room may negatively affect infection risk [20]; and (2) emergency surgeries are usually assigned to junior residents and performed past elective hours; hence, surgeon expertise and efficiency may also affect infection risk [21]. In addition, we suggest that contributory factors may include reduced preoperative preparation compared to elective cases due to the urgent nature of the surgery, as well as the poorer clinical condition of patients operated on an emergency basis.

Surgeries performed by junior residents carried 7.2 times higher odds of shunt infection than those performed by senior residents, possibly reflecting the difference in operative efficiency and technical familiarity. This was further supported by studies showing that a surgeon’s experience impacts postoperative outcomes [22].

In our study, endoscopic procedures were associated with a non-significant, 6.4-fold higher odds of shunt infection compared to non-endoscopic procedures. McGirt et al. [15] reported a 1.6-fold increase in shunt infection risk with the use of an endoscope. They attributed this to an additional increase in operative time in complex types of hydrocephalus [15]. Kontny et al. [23] showed a higher shunt infection rate of 13.6% in procedures lasting more than 90 minutes compared to 5.2% in procedures lasting 30 minutes or less. Furthermore, results from different studies support that a decrease in operative time decreases the risk of shunt infection [7,23,24]; hence, endoscopy should be reserved for cases with a clear and specific indication.

Although we found no association between OR personnel count and infection, other studies have suggested thresholds of ≤4 or ≤7 people to reduce contamination [13]. A larger number of personnel in the OR may serve as an increased source of infection through contamination [7]. In addition, OR personnel turnover, especially during break time and change of shifts, increased door events, which increases bacterial air contamination, in turn, increasing the risk of infection [25].

Protocol compliance

In our study, full compliance with the shunt protocol did not demonstrate a statistically significant decrease in shunt infection risk compared to cases with only partial compliance. On the other hand, numerous studies have reported that simply following a perioperative protocol, regardless of the number of steps, decreases shunt infection risk [26]. On further analysis of the different protocol steps, application of Ioban seemed to have a protective effect on shunt infection risk (OR = 0.24, 95% CI = 0.05, 1.12], p = 0.070), although it was non-significant. Ioban has been shown to decrease shunt infection risk in different studies; however, further comparison with other materials and practices is needed [27]. Low compliance rate to the shunt protocol in our study can be attributed to the large number of emergency cases, where full body bath and shampooing could not be done in the emergency room. In addition, staffing limitations of the nurses force them to handle multiple cases at a time, limiting door traffic. Lastly, surgeon preference is also a factor in the completion of the shunt protocol, as some surgeons do not prefer using Ioban.

Low- and middle-income countries

Our shunt infection rates were similar to the results of studies done in LMICs [3]. Factors that were found to be significant in increasing the odds of shunt infection in LMIC studies were age, post-infective etiology, and poor nutritional status [3]. Because of the high infection rate in LMICs compared to HIC counterparts, perioperative protocols were also instituted and adapted to their resource-limited setting. These were shown to improve shunt infection rates, confirming that the use of a perioperative protocol reduces the risk of infection [28]. The HCRN developed the five core steps, which further simplified the protocol to evidence-based steps, emphasizing adherence to the protocol irrespective of the number of steps [10]. Further studies regarding the use of this abbreviated protocol are yet to be performed in LMICs to assess its adaptability and impact on shunt infection risk.

Aside from the stark difference in shunt infection rates between HICs and LMICs, patient characteristics and perioperative factors are also different. Causes of shunt infection in HICs are usually due to prematurity and post-hemorrhagic hydrocephalus, while post-infectious and myelomeningocele-associated hydrocephalus predominate in LMICs [3]. Programmable and antibiotic-impregnated shunts are readily available in HICs but are less accessible in LMICs. Hygiene practices, especially in rural areas, poor maternal health [3], nutritional status [24], treatment delays [29], and other socioeconomic factors [30] impact management, follow-up, and outcomes of shunt surgery.

In our institution, the relatively high shunt infection rate may be attributed to several factors, such as the predominance of patients less than a year old, the greater proportion of procedures performed by junior neurosurgical trainees, and the high frequency of congenital pathologies, which are inherently associated with increased shunt infection. In addition, poor compliance with the shunt protocol may also be contributory.

Limitations and recommendations

The main limitation of this study was the small sample size. The power of the study and generalizability can be improved by enrolling a larger cohort of patients. Collaboration with other institutions to implement this protocol can also be done to further strengthen the results of the study and facilitate the creation of national guidelines. Revising and simplifying the shunt protocol by reducing the number of steps may help improve compliance. Strict adherence to the shunt protocol should be enforced among all OR personnel, with the goal of continued implementation to embed its core principles into routine practice.

## Conclusions

Full compliance with the shunt protocol was observed in only 52% of shunt operations. Given the suboptimal compliance, we were unable to draw any significant conclusions regarding its efficacy. There were no factors identified among the patient characteristics and operative variables that were found to be associated with shunt infection risk. Larger prospective studies and greater adherence to the shunt protocol are recommended.
